# Cytokine-induced killer cells co-cultured with dendritic cells loaded with the protein lysate produced by radiofrequency ablation induce a specific antitumor response

**DOI:** 10.3892/ol.2015.2977

**Published:** 2015-02-17

**Authors:** CHAN-CHAN SHAN, LIANG-RONG SHI, MEI-QIAN DING, YI-BEI ZHU, XIAO-DONG LI, BIN XU, JING-TING JIANG, CHANG-PING WU

**Affiliations:** 1 Department of Tumor Biological Treatment, The Third Affiliated Hospital of Soochow University, Changzhou, P.R. China; 2 Department of Oncology, The Third Affiliated Hospital of Soochow University, Changzhou, P.R. China; 3 Institute of Biotechnology, Soochow University, Suzhou, Jiangsu, P.R. China

**Keywords:** thermal ablation, dendritic cells, cytokine-induced killer cells, colon, antigen load, protein lysate

## Abstract

Radiofrequency ablation (RFA) causes coagulative necrosis of tumor tissue and the production of local tumor protein debris. These fragments of tumor protein debris contain a large number of various antigens, which can stimulate a specific cellular immune response. In the present study, dendritic cells (DCs) were loaded with tumor protein lysate antigens that were produced *in situ* by RFA, and were used to treat murine colon carcinoma in combination with cytokine-induced killer (CIK) cells. Subsequent to the treatment of murine colon carcinoma by RFA, the *in situ* supernatant of tumor lysis was collected and the DCs were loaded with the lysate antigen to generate Ag-DCs. CIK cells induced from the spleen cells of mice were co-cultured with Ag-DCs to generate Ag-DC-CIK cells. The results revealed that the Ag-DC-CIK cells exhibited strong antitumor activity *in vitro* and *in vivo*. The morphology and immunophenotypes of these cells were determined using microscopy and flow cytometry, respectively. The cytotoxic activity of Ag-DC-CIK cells was determined using a CCK-8 assay. To establish a mouse model, mice were randomized into Ag-DC-CIK, DC-CIK, CIK and PBS control groups and monitored for tumor growth and survival time. ANOVA was used to compare the trends in the three groups for implanted tumor volumes. The log-rank test was used to compare the survival time. The present findings indicated that DCs loaded with the protein lysate antigens of tumors, produced *in situ* by RFA, combined with CIK cells may be a novel strategy for cancer treatment.

## Introduction

Thermal ablation is a novel minimally invasive treatment method that is mainly performed using radio frequency, microwave or laser-generated heat, resulting in local coagulative tumor necrosis. Radiofrequency thermal ablation techniques mainly include microwaves, freezing and focused ultrasound, and have been increasingly used for the local treatment of unresectable malignant invasive lesions (1). Radiofrequency ablation (RFA) is a type of thermal ablation with potential as a minimally invasive treatment following the surgical resection of tumors, and has been recommended as the standard treatment for primary liver cancer and as a localized treatment for unresectable non-small cell lung cancer, according to the National Comprehensive Cancer Network guidelines (2). Local thermal ablation results in minor trauma and fewer complications when compared with surgery. The patients also recover faster than patients treated with surgery and are satisfied with the resulting quality of life, and the procedure can be repeated several times. Currently, RFA has been successfully used to treat lung, breast, kidney, liver and bone malignancies (3–7).

Dendritic cells (DCs) are often termed nature’s adjuvants and have become agents for antigen delivery. The antigen-presenting ability of DCs is 1,000 times higher compared with the ability of normal antigen-presenting cells (APCs). DCs are the only APC that can activate early T cells, and are therefore at the center of the immune response. Through DCs, induced antitumor immunity has become an important method for tumor immunotherapy (8). DCs are able to activate naive cluster of differentiation (CD)4^+^ T helper cells and unprimed CD8^+^ cytotoxic T lymphocytes (CTLs). DC-based regimens have been used to produce tumor-specific antigen-presenting cells and to generate CTL responses against cancer cells (9,10). The aim of DC vaccination is to induce tumor-specific effector T cells that can specifically reduce the tumor mass and induce immunological memory to control tumor recurrence (11). Previous studies have found that RFA not only results in coagulative tumor necrosis, but also initiates a series of biological effects through the local high temperature surrounding the tumor (12,13). The induction of tumor necrosis increases the quantity of heat shock protein presented as a tumor antigen, which activates DCs to a certain extent and stimulates the proliferation of CD4^+^ T cells in the peripheral blood, therefore improving the body’s immune status and enhancing the antitumor immunity (12).

Cytokine-induced killer (CIK) cells are a unique population of CTLs with a characteristic CD3^+^/CD56^+^ phenotype (14). CIK cells not only demonstrate strong antitumor activity that is similar to T cells, but also exhibit non-major histocompatibility complex (MHC)-restricted cytotoxicity, similar to natural killer (NK) cells (15). CIK cells demonstrate high tumor cytotoxic activity, a broad spectrum of tumor targets and low toxicity to normal tissues *in vitro*, which allows for wide use in current clinical adoptive immunotherapy (16).

Animal model experiments confirmed that the number of tumor metastases decreased subsequent to thermal ablation, and the occurrence of novel tumors also decreased (17). However, RFA-induced antitumor immunity is insufficient to control metastatic progression (18,19). On tumor ablation *in situ*, large amounts of tumor debris are released that may potentially be taken up by the immune system (20,21). It has previously been investigated whether ablated tumor debris induces a systemic immune response, and while systematic analysis continues to be reported, convincing evidence is lacking (22). Therefore, it is of considerable importance to investigate significant amplification methods for antitumor immune specific effects that further improve tumor control based on *in situ* antigen exposure subsequent to ablation. The ability of DCs loaded with protein lysate antigen produced *in situ* by RFA to enhance the antitumor activity of CIK cells has not yet been reported. In the present study, a tumor-bearing mouse model was established, and the *in situ* tumor lysate produced by RFA was obtained. The DCs were loaded with the antigen *in vitro* and were co-cultured with CIK cells. Through phenotypical analysis by flow cytometry, analysis of the cytotoxic activity *in vitro* using a cell counting kit-8 (CCK-8) kit assay and the establishment of a cancer prevention and treatment mouse model, the present study aimed to demonstrate the antitumor activity of DC-CIK cells loaded with lysate produced by RFA, and also aimed to provide a theoretical basis for the treatment mode of tumors using RFA combined with immune therapy.

## Materials and methods

### Animal and tumor model

Male BALB/C mice were purchased at 6–7 weeks of age, weighing 18–22 g, from the Comparative Medicine Centre of Yangzhou University (Yangzhou, Jiangsu, China). All animals were housed under standard conditions in accordance with the animal care and use committee of Soochow University (Suzhou, China), which follows the China Public Health Service’s guide for care and use of animals (23). This study was approved by the ethics committee of Soochow University.

The present study used murine colon carcinoma C26 cells (Chinese Academy of Sciences Cell Bank, Shanghai, China). The C26 cells were cultured in RPMI 1640 (Gibco Life Technologies, Carlsbad, CA, USA) that contained 10% fetal calf serum (Gibco Life Technologies), 10% L-glutamine, 0.5% penicillin/streptomycin, 10% nonessential amino acids and 10% pyruvate, in a 5% CO_2_ atmosphere and incubated at 37°C. The backs of the mice were completely shaved, and a subcutaneous (SC) injection that contained 5×10^5^ C26 cells was administered to the back. Mice were selected for ablation when the diameter of the tumor ranged between 5–7 mm and was approximately round in shape.

### In vitro differentiation of DCs from bone marrow (BM) precursors

BM-derived DCs were generated from primary cultures of femoral marrow obtained from 6–8-week-old male BALB/C mice, as described above. The DCs were derived from BM as previously described (24). Briefly, the BM cells were depleted of lymphocytes. The remaining cells were cultured in RPMI 1640 supplemented with 10% fetal bovine serum (FBS; Gibco), 1% HEPES buffer, 1% penicillin-streptomycin, 1% non-essential amino acids, 1% sodium pyruvate, 1% L-glutamine, 2-mercaptoethanol, recombinant murine granulocyte–macrophage colony-stimulating factor (rmGM-CSF; 20 ng/ml; R&D Systems, Inc., Minneapolis, MA, USA) and recombinant murine interleukin 4 (rmIL-4; 10 ng/ml; R&D Systems). The media was changed every two days, and fresh complete RPMI medium containing rmGM-CSF and rmIL-4 was applied each time. On day seven, tumor necrosis factor-α (TNF-α; 10 ng/ml; R&D Systems) was added to the media. The change in the shape, size, number, distribution and adherence to the glass were observed under an inverted microscope (Olympus CKX31; Olympus Corporation, Tokyo, Japan). Subsequent to an *ex vivo* culture, the percentages of differentiated DCs were determined by fluorescence activated cell sorting (FACS) analysis of the expression of cluster of differentiation (CD)11c, CD80, CD86 and MHC II.

### RF ablation procedure

The C26 tumor-bearing mice were anesthetized by 10% chloral hydrate (0.004 ml/g) and the tumor area was shaved and disinfected with alcohol. The mice were positioned prone on an electricity-conducting grounding pad (AngioDynamics, Inc., Latham, NY, USA). The ablation was performed using a monopolar radiofrequency needle (17G; AngioDynamics, Latham, NY, USA) with a 4-mm active tip that was percutaneously inserted orthogonal to the skin in the center of the tumor. The impedance and tumor temperature were measured using a 50-W RF lesion generator (RITA Model 1500x; AngioDynamics). The mice with tumors 50–200 mm^3^ in size were subjected to RFA using two consecutive treatment cycles of 120 sec with the power limited to 7 W and temperature limited to 70°C. The dose of RFA varied according to the different exposure times until the temperature in the center of the tumor was raised to 57°C. Subsequent to RFA, the treatment area became hot. The mice were returned to the cage, and were maintained at a constant temperature until they were sober.

### Tumor lysate preparation

Within 24 h following RFA, the ablation tissue was removed under sterile conditions. The tissue was added to a petri dish containing phosphate-buffered saline (PBS) prior to being sliced and ground until the tissue was a homogeneous slurry liquid. The resulting liquid was frozen in liquid nitrogen for 10 min and then thawed in a 37° water bath for 10 min. The freeze-thaw cycle was repeated five times in rapid succession, and the liquid was then centrifuged at 9,600 × g for 2 min. Finally, the supernatant was collected and sterilized through a 0.22 μm filter membrane. The protein content of the tumor lysate was determined using a Lowry protein assay kit, according to the manufacturer’s instructions (Bio-Rad). The samples were aliquoted and stored in liquid nitrogen until use.

### In vitro differentiation of CIK from spleen precursors

The mice were sacrificed by cervical dislocation and were immediately immersed in the volume fraction of 75% ethanol for 5 min. The spleen was removed under sterile surgical conditions and the spleen cells were isolated. The cells were washed twice with RPMI 1640 containing 1% heat-inactivated FBS. The concentration of cells was adjusted to 1×10^6^ cell/ml in 24-well plates prior to being resuspended in RPMI 1640, supplemented with 10% FBS, and cultured at 37°C in a 5% CO_2_ atmosphere. On the same day, interferon-γ (IFN-γ; 1,000 U/ml; BioLegend, Inc., San Diego, CA, USA) was added. Anti-CD3 monoclonal antibody (1 μg/ml; R&D Systems), CD28 (1 μg/ml; BD Biosciences, Franklin Lakes, NJ, USA), IL-1 (100 U/ml, R&D Systems), IL-2 (300 U/ml, R&D Systems) were added after 24 h. Fresh medium and IL-2 were added to each well every two days.

### Ag pulsing of DCs and co-culture with CIK cells

Subsequent to five days of culture, the DCs were divided into two groups. One group was termed the Ag-DC group, comprising cells supplemented with the antigen, as aforementioned, at a final concentration of 5 μg/ml. The other group was termed the DC group, which comprised cells without the antigen, and acted as a control group. The two cell groups were incubated at 37°C in a 5% CO_2_ atmosphere for 48 h. On day nine, these two groups of cells were co-cultured with CIK cells, which had been cultured for seven days, at a cell ratio of 1:5, for 48 h.

### Flow cytometry

Single-cell suspensions of BM-derived DCs and spleen-derived CIK cells that had been cultured for seven days, as aforementioned, were analyzed by FACScan flow cytometry (Cytomics FC500 MPL; Beckman Coulter, Brea, CA, USA). The density of cells was adjusted to 1×10^5^ cells/ml. The IgG isotype control antibody was set as a control. The expression of surface makers in DCs were analyzed, consisting of anti-mouse MHC II (I-A/I-E)-fluorescein isothiocyanate (FITC), anti-mouse CD11c-phycoerythrin (PE), anti-mouse CD80 (B7-1)-PE and anti-mouse CD86 (B7-2)-FITC. Flow cytometry was used to detect differences in the positive rate of molecules on the surface of DCs and Ag-DCs. Anti-mouse CD3-FITC and anti-mouse NK antigen 1.1 (NK1.1)-PE were used to identify CIK cells. All detection antibodies were obtained from eBioscience (San Diego, CA, USA), and the dose of antibodies was in strict accordance with the supplier’s instructions. The DCs, Ag-DCs and CIK cells were suspended in PBS containing 1% bovine serum albumin and were stained with various fluorochrome-conjugated monoclonal antibodies (mAbs) for 30 min on ice. Subsequent to staining, the cells were washed twice with FACS buffer and stored in PBS containing 2% paraformaldehyde until FACS analysis was performed. Flow cytometric analysis was performed using FlowJo software (FlowJo, LLC, Ashland, OR, USA).

### In vitro antitumor activity analysis

The *in vitro* cytotoxicity of the Ag-DC-CIK, DC-CIK and CIK cells against the C26 cells was determined using a CCK8 kit (Dojindo Molecular Technologies, Inc., Kumamoto, Japan). The DC-CIK, Ag-DC-CIK and CIK cells were harvested 11 days after induction and were used as effector cells. The murine colon carcinoma C26 cells were used as target cells. The effector and target cells were added to 96-well plates at a ratio of 5:1, 10:1 or 20:1. The cells were incubated for 24 h at 37°C in a 5% CO_2_ atmosphere. The groups that comprised a mixture of cell types were the experimental groups, while the control groups contained only one cell type of the C26, CIK, Ag-DC-CIK or DC-CIK cells, or 1640-RPMI cultivating solution. The effector-target ratios were 5:1, 10:1 or 20:1. The CCK8 assay was performed to evaluate cell viability, and optical density (OD) was read at 570 nm. The assays were performed in triplicate and the cytotoxic activity was calculated as follows:

$$\text{Cytotoxic activity},\;\%=[1-({\text{OD}}_{\text{effector and target cells}}-{\text{OD}}_{\text{effector cells}})/{\text{OD}}_{\text{target cells}}]\times 100.$$

### In vivo antitumor activity analysis

The BALB/C mice were randomly divided into four groups, the Ag-DC-CIK (n=5), DC-CIK (n=5), CIK (n=5) and PBS control (n=5) groups. Various sections of the mice were marked with 5% picric acid. The backs of the mice were completely shaved, and a SC injection containing 5×10^5^ C26 cells/mouse was then administered to the back. After three days, 1.5×10^6^ Ag-DC-CIKs, DC-CIKs or CIK cells in 0.2 ml PBS were intravenously injected into the tail of the mice in the appropriate group.

BALB/C mice were randomly divided into four groups as aforementioned. In total, 1.5×10^6^ Ag-DC-CIK, DC-CIK or CIK cells in 0.2 ml PBS, or 0.2 ml PBS, were administered intravenously into the tail of the mice in the appropriate group. After three days, 5×10^5^ C26 cells/mouse were administered through a SC injection to the backs of the mice in the four groups.

The mice were monitored daily, and the appearance of a hard texture at the vaccination site was identified as a tumor nodule. The tumor volumes were measured every other day using digital calipers (Wuxi Xigong Tools & Measuring Instruments Co., Ltd., Wuxi, China) and were calculated using the formula (A × B^2^) × 0.5, in which A was the largest and B was the shortest dimension. When the largest diameter of the tumor reached 2 cm or the mice experienced severe ulcers, the mice were sacrificed.

### Statistical analysis

GraphPad Prism 5 software (GraphPad Software, Inc., La Jolla, CA, USA) was used for the statistical analysis. The results were presented as the mean ± standard error of the mean. The means of various treatment groups were compared using two-way analysis of variance. P<0.05 was considered to indicate a statistically significant difference. The survival estimations were determined using the Kaplan and Meier method.

## Results

### Morphology of DCs and CIK cells

**Figure 1:**
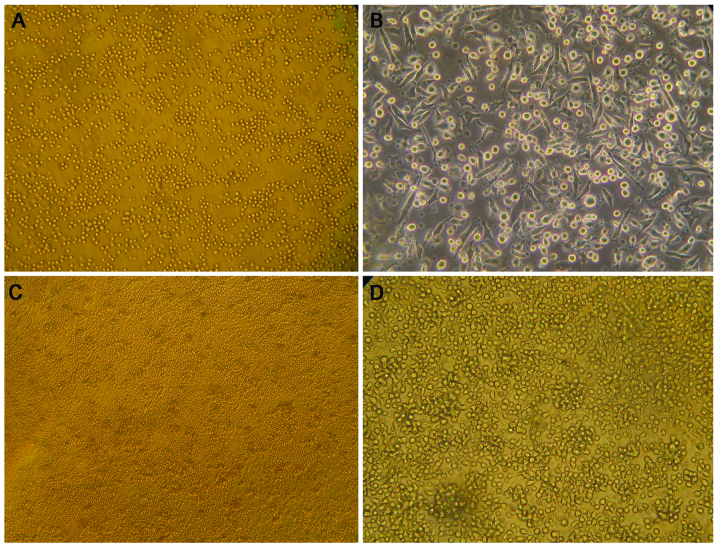
The morphology of DCs and cytokine-induced killer cells under a microsope. (A) The primitive cells from mouse bone marrow were mainly small and round cells (phase contrast microscope; magnification, ×40). (B) Subsequent to nine days of induction, a large number of typical DCs released from the colony. The cells were thriving, with evident thick and long dendritic protrusions (phase contrast microscope; magnification, ×100). (C) Subsequent to induction for three days, the primitive cells of the slpeen from BALB/C mice appeared large, certain cells adhered to the bottom of the plate and a small colony had formed (phase contrast microscope; magnification, ×40). (D) On post-induction day 10, the cells entered the stationary phase of growth and became a more regular shape. As the culture time increased, the proliferation of cells slowed down, the shape was irregular, and necrosis fragmentation of the cells and cell apoptosis increased (phase contrast microscope; magnification, ×100). DCs, dendritic cells.

The separation and purification of mouse BM cells and observation by microscopy revealed the cells to be mainly small and round in shape (Fig. 1A). Subsequent to being cultured for 24 h, half the adherent cells were visible at the bottom of the plate, a small number of colonies had formed and the cells were small and round, without evident protrusions. On day three, the number of adherent cells and colonies increased, and the cells became larger, circular or elliptical in shape. On day five, the cells were released from colonies, the cells became larger, and exhibited the irregular, fusiform or stellate shape that is characteristic of immature DCs. On day seven, the DCs of the no-antigen group increased in size and the shape tended to be a typical star. The DCs of the antigen group possessed between two and five prominences and the cells were congregated. On day nine, a large number of typical DCs were released from the colony. The DCs were thriving, with evident thick and long dendritic protrusions (Fig. 1B). On average, 5×10^7^ BM cells were separated by each mouse, and ~8×10^6^ DCs could be obtained following induction.

Primitive spleen cells were activated subsequent to the addition of IFN-γ on day one, and the cells continued to activate following the addition of IL-1, IL-2, CD3McAb and CD28. The cells appeared large, certain cells were adherent to the bottom of the plate and small colonies had formed (Fig. 1C). On day three, the number of colonies had markedly increased and the cells had proliferated. On day seven, the suspended cells had become round or oval-shaped, similar to typical small lymphocytic cells. The cells entered the stationary phase of growth 10 days following induction and became more regular in shape. The proliferation of cells decreased, the shape was irregular and necrotic fragmentation of the cells and cell apoptosis increased with the culture time (Fig. 1D).

### Immunophenotypical analysis

**Figure 2:**
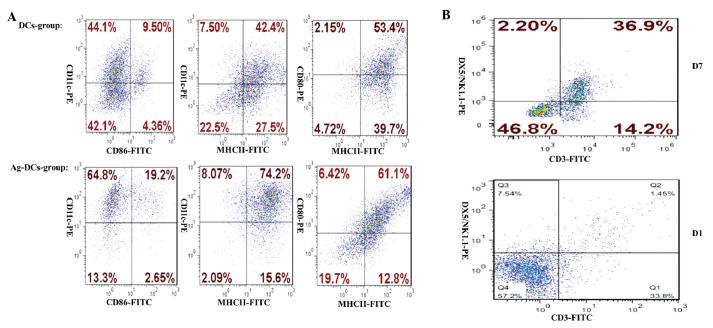
Immunophenotype of DCs, Ag-DCs and CIK cells. (A) Subsequent to seven days of induction, the proportion of CD86^+^/CD11c^+^, MHC II^+^/CD80^+^ and MHC II^+^/CD80^+^ cells in DCs was 9.50, 42.4 and 53.4%, respectively, while in Ag-DCs, the proportions were higher, consisting of 19.2%, 74.2 and 61.1%, respectively. (B) On the first day of CIK cell culture, the percentage of CD3^+^/NK1.1^+^ cells was 1.45%, and subsequent to seven days of induction, the percentage of CD3^+^/NK1.1^+^ cells was increased to 36.9%. DCs, dendritic cells; Ag-DCs, DCs supplemented with tumor antigen; CIK, cytokine-induced killer; CD, cluster of differentiation; MHC, major histocompatibility complex; NK1.1, natural killer antigen 1.1; PE, phycoerythrin; FITC, fluorescein isothiocyanate.

The proportion of CD86^+^/CD11c^+^, MHC II^+^/CD11c^+^ and MHC II^+^/CD80^+^ cells in the DC population was 9.50, 42.4 and 53.4%, respectively, while in the Ag-DC population, the proportions were 19.2, 74.2 and 61.1%, respectively (Fig. 2A). On the first day of CIK cell culture, the percentage of CD3^+^/NK1.1^+^ cells was 1.45%, and subsequent to the induction of CIK cells for seven days, the percentage of CD3^+^/NK1.1^+^ cells had increased to 36.9% (Fig. 2B).

### Comparison of the cytotoxicity of effector cells in vitro

Using the CCK-8 assay, it was revealed that Ag-DC-CIK cells exhibited the highest cytotoxic activity against C26 cells. When the ratio of effector cells to target cells was 5:1, the cytotoxic activity against C26 cells was 74.9±3.5% in the Ag-DC-CIK cells, 71.2±2.1% in the DC-CIK cells and 68.7±2.9% in the CIK cells was statistically significant (P=0.007). When the ratio of effector cells to target cells was 10:1, the cytotoxic activity against C26 cells was 82.3±4.5% in Ag-DC-CIK cells, 77.1±5.1% in the DC-CIK cells and 72.7±2.8% in the CIK cells it was statistically significant (P=0.005). When the ratio of effector cells to target cells was 20:1, the cytotoxic activity against C26 cells was 83.2±1.9% in the Ag-DC-CIK cells, 77.2±4.2% in the DC-CIK cells and 73±2.6% in the CIK cells it was statisticaly significant (P=0.0006) (Table I).

### In vivo antitumor activity analysis

**Figure 3:**
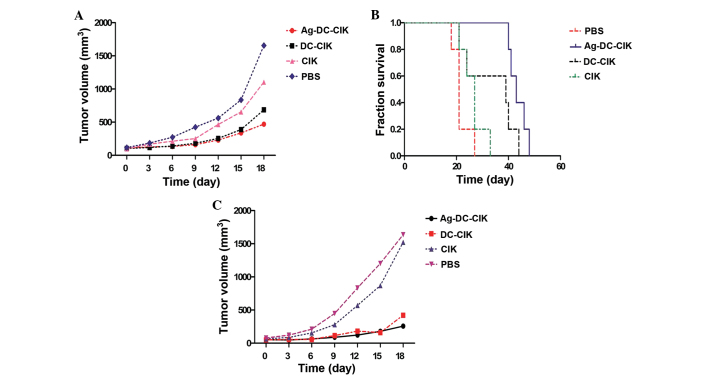
Tumor growth curve and comparison between survival time of the groups. The BALB/C mice were randomly divided into four groups, the Ag-DC-CIK, DC-CIK, CIK and PBS control groups. The mice were completely shaved at the back, and were subcutaneously injected at the back with 5×10^5^ C26 cells/mouse. After three days, the Ag-DC-CIK, DC-CIK, CIK and PBS groups were administered with 1.5×10^6^/0.2 ml Ag-DC-CIK, DC-CIK or CIK cells, or 0.2 ml PBS, respectively, through an intravenous injection to the tail. (A) The tumor volume in the Ag-DC-CIK group was smaller compared with the DC-CIK, CIK and the PBS control groups. The difference was statistically significant (F_1_=69.90; F_2_=33,71; P<0.00). (B) The average survival period of the Ag-DC-CIK group was 44±3 days, while the DC-CIK, CIK and PBS control groups exhibited survival times of 34±10, 26±4 and 22±3 days, respectively. The difference was statistically significant (P=0.01). The BALB/C mice were randomly divided into four groups, comprising the Ag-DC-CIK (n=5), DC-CIK (n=5), CIK (n=5) and PBS control groups (n=5). 1.5×10^6^/0.2 ml Ag-DC-CIK, DC-CIK or CIK cells, or 0.2 ml PBS were respectively intravenously injected to the tail of the four groups of mice. Three days later, 5×10^5^ C26 cells/mouse were subcutaneously injected to the back of the four groups of mice. (C) The tumor volume of the Ag-DC-CIK group was smaller compared with the DC-CIK, CIK and PBS control groups, and the difference was statistically significant (F_1_=165.48; F_2_=151.54; P<0.00). DCs, dendritic cells; CIK, cytokine-induced killer; Ag-DC-CIK, DCs supplemented with tumor antigen and co-cultured with CIK cells; DC-CIK, DCs co-cultured with CIK cells; PBS, phosphate-buffered saline.

The tumor volume of the Ag-DC-CIK group was significantly smaller compared with the DC-CIK, CIK and PBS control groups (F_1_=69.90; F_2_=33.71; P<0.0001; Fig. 3A). The average survival periods of the Ag-DC-CIK, DC-CIK, CIK and PBS control cells were 44±3, 34±10, 26±4 and 22±3 days, respectively, and the difference between the average survival periods of all four groups was statistically significant (P=0.01; Fig. 3B).

Seven days after the four groups of mice were injected with C26 cells, the tumor formation rate of the Ag-DC-CIK group was 60% (three out of five mice), the DC-CIK group was 80% (four out of five mice), the CIK group was 80% (four out of five mice), and the PBS control group was 100% (five out of five mice). The tumor volume of the Ag-DC-CIK group was significantly smaller compared with the DC-CIK, CIK and PBS control groups (F_1_=165.48; F_2_=151.54: P<0.0001; Fig. 3C).

## Discussion

Despite RFA being a minimally invasive technique, it can be used as an alternative to surgical resection (1,3). Previous studies have reported that RFA is not superior to surgical resection in the management of tumor recurrence, indicating that the immune responses elicited by RFA alone are not sufficient to prevent tumor progression (25,26). The *in situ* residual tumor tissue debris produced by RFA and the tumor antigen exposure promote tumor infiltration through lymphocytes, to induce a specific antitumor immune response (27). However, due to the presence of immunosuppressive factors, the immune response is stimulated at a low level, which is insufficient to prevent tumor progression (28). The immune response requires amplification to control tumor progression subsequent to ablation by adjuvants or immune cells (29–31).

Thermal ablation and other minimally invasive therapies that are combined with CIK cells for the treatment of liver cancer models have been clinically applied. It has been reported that minimally invasive treatment of complete remission was obtained subsequent to RFA and sequential CIK cell therapy, which may delay tumor recurrence and prolong the overall survival of patients. A number of studies have investigated minimally invasive techniques combined with CIK cells as a treatment for primary liver cancer, which was the initial description for the basis of minimally invasive techniques for the majority of tumors, and the use of CIK cells for the further removal of small lesions delays the progress of the tumor (32–34). However, previous research and clinical applications sequentially apply the two treatment methods, ignoring an inherent association that may exist between the two and produce immune stimulation. Thermal ablation is simultaneously a physical and immune therapy. RFA produces coagulative necrosis tissue and local tumor cell debris, which contains a large quantity of tumor antigen that can be identified to stimulate a specific cellular immune response.

The total protein of tumor cells includes abundant membrane antigens, MHC I and II epitopes and various heat shock proteins. Subsequent to the proteins being taken up, processed and presented by DCs, the proteins can induce the cloning of multiple tumor antigens, and effectively induce specific CTLs against the antigen to exert antitumor immune activity. The number and activity of tumor-specific T lymphocytes are the major determinants of anti-neoplastic immunity. Although the CIK cells possess the antitumor activity of T lymphocytes and the non-MHC restricted antitumor characteristics of NK cells, the CIK cells do not express Fc receptor and are unable to produce antibody-dependent cytotoxicity (35,36). Co-culture of DCs and CIK cells results in not only an ability to participate in non-MHC restricted CIK cell cytotoxicity, but also an ability to stimulate antigen-loaded DCs to mediate MHC-restricted cytotoxic effects, which enhance the specific cytotoxic effect on the specific target cells. Previous studies have found that DC-CIK cells possess strong proliferative activity and cytotoxicity, and lower clinical side-effects (37–39). Subsequent to the induction of DCs, CD3^+^/CD56^+^ double-positive cells and CD8^+^ T cells were significantly increased in the CIK cell population (40). The inhibition of T cell immunity, namely CD4^+^/CD25^+^ Treg cells, was significantly reduced, thereby weakening the antitumor immune efficiency of Treg cells *in vitro* (41,42). The present study also confirmed that DCs and CIK cells demonstrate a synergistic effect. Subsequent to being co-cultured, the expression of costimulatory molecules on the surface of DCs and the antigen presenting capacity of DCs significantly increased. In addition, the proliferation and cytotoxic activity of CIK cells *in vitro* and *in vivo* can be enhanced. The ablation *in situ* tissue lysate can provide effective antigens, and the stimulation of the antigen can make the synergistic effect more evident. Possible mechanisms of the enhancement of antitumor activity may involve the input of CIK cells, which can exert antitumor activity, or DCs, which can secrete a variety of cytokines that promote the activation of NK cells *in vivo*, including IL-2, IL-12 and IFN-γ.

The present data indicate that DCs capture RFA protein lysate antigen *in situ*. These cells highly express molecules including CD80, CD86 and MHC II, and the *in vitro* cytotoxic activity was found to be significantly higher in the DCs that capture the antigen compared with the general DCs. As the mouse experiments demonstrated, an ablation lysate-pulsed DC vaccine combined with CIK cells exhibits strong advantages in tumor therapy and prevention. Overall, DCs loaded with the products of *in situ* thermal tumor ablation, administered as a DC vaccine combined with CIK cells, demonstrates improved cytotoxic activity.

In the present study, the process of RFA to produce residual tumor necrotic debris, which then underwent repeated freezing and thawing to obtain whole cell lysates for use as an antigen, was simple, and a comprehensive tumor antigen can be obtained without requiring specific antigenic epitopes to ensure comprehensive and powerful tumor immunity. The antigen is readily available in the clinic, with a high clinical utility, but the specific clinical standardization of clinical trials remains to be confirmed. In conclusion, the present results provide a theoretical basis for the optimization of immunotherapy combined with RFA, which may be of great significance for the prevention of tumor recurrence subsequent to RFA.

## Figures and Tables

**Table I:** Cytotoxicity of Ag-DC-CIKs, DC-CIKs and CIK cells against C26 cells.

	Cytotoxicity, %		
	
Group	Effector/target cell Ratio 5:1	Effector/target cell Ratio 10:1	Effector/target cell Ratio 20:1
Ag-DC-CIK	74.9±3.5^a^,^b^	82.3±4.5^a^,^b^	83.2±1.9^a^,^b^
DC-CIK	71.2±2.1	77.1±5.1^c^	77.2±4.2^c^
CIK	68.7±2.9	72.7±2.8	73.0±2.6

Values are provided as the mean ± standard deviation. When the ratio of effector cells to target cells was 5:1, it was statistically significant (P=0.007). When the ratio of effector cells to target cells was 10:1, it was statistically significant (P=0.005).

a P<0.05 vs. DC-CIK group;

b P<0.001 vs. CIK group;

c P<0.05 vs. CIK group.

CIK, cytokine-induced killer; DC-CIK, dendritic cells co-cultured with CIK cells; Ag-DC-CIK, DCs supplemented with tumor antigen and co-cultured with CIK cells.

## References

[1] Pathak S, Jones R, Tang JM (2011). Ablative therapies for colorectal liver metastases: a systematic review. Colorectal Dis.

[2] NCCN Guidelines for Patients (2014). Non-Small Cell Lung Cancer Version 1.

[3] Gervais DA, Goldberg SN, Brown DB, Soulen MC, Millward SF, Rajan DK (2009). Society of Interventional Radiology position statement on percutaneous radiofrequency ablation for the treatment of liver tumors. J Vasc Interv Radiol.

[4] Schneider T, Heussel CP, Herth FJ, Dienemann H (2013). Thermal ablation of malignant lung tumors. Dtsch Arztebl Int.

[5] Vogl TJ, Zegelman A, Bechstein WO, Zeuzem S, Zangos S (2013). Treatment of liver metastases of colorectal carcinoma: overview of hyperthermal ablation methods. Dtsch Med Wochenschr.

[6] Olweny EO, Cadeddu JA (2012). Novel methods for renal tissue ablation. Curr Opin Urol.

[7] Alvarado R, Mahon B, Valadez C (2009). Thermal ablation of the goat mammary gland as a model for post-lumpectomy treatment of breast cancer: preliminary observations. Int J Hyperthermia.

[8] Cui Y, Yang X, Zhu W, Li J, Wu X, Pang Y (2013). Immune response, clinical outcome and safety of dendritic cell vaccine in combination with cytokine-induced killer cell therapy in cancer patients. Oncol Lett.

[9] Kalinski P, Okada H (2010). Polarized dendritic cells as cancer vaccines: directing effector-type T cells to tumors. Semin Immunol.

[10] Steinman RM (2012). Decisions about dendritic cells: past, present, and future. Annu Rev Immunol.

[11] Palucka K, Banchereau J (2012). Cancer immunotherapy via dendritic cells. Nat Rev Cancer.

[12] Liu Q, Zhai B, Yang W (2009). Abrogation of local cancer recurrence after radiofrequency ablation by dendritic cell-based hyperthermic tumor vaccine. Mol Ther.

[13] Nikfarjam M, Muralidharan V, Su K (2005). Patterns of heat shock protein (HSP70) expression and Kupffer cell activity following thermal ablation of liver and colorectal liver metastases. Int J Hyperthermia.

[14] Schmidt-Wolf IG, Negrin RS, Kiem HP (1991). Use of a SCID mouse/human lymphoma model to evaluate cytokine-induced killer cells with potent antitumor cell activity. J Exp Med.

[15] Linn YC, Hui KM (2010). Cytokine-induced NK-like T cells: from bench to bedside. J Biomed Biotechnol.

[16] Jiang JT, Shen YP, Wu CP (2010). Increasing the frequency of CIK cells adoptive immunotherapy may decrease risk of death in gastric cancer patients. World J Gastroenterol.

[17] Idia N, Nakamoto Y, Baba T (2010). Antitumor effect after radiofrequency ablation of murine hepatoma is augmented by an active variant of CC Chemokine ligand 3/macrophage inflammatory protein-1alpha. Cancer Res.

[18] Julianov AE, Karashmalakov AG (2011). Percutaneous radiofrequency ablation as first-line treatment in patients with early colorectal liver metastases amenable to surgery: is it justified?. Ann Surg.

[19] Mulier S, Ni Y, Jamart J (2008). Radiofrequency ablation versus resection for resectable colorectal liver metastases: time for a randomized trial?. Ann Surg Oncol.

[20] Haen SP, Pereira PL, Salih HR (2011). More than just tumor destruction: immunomodulation by thermal ablation of cancer. Clin Dev Immunol.

[21] Widenmeyer M, Shebzukhov Y, Haen SP (2011). Analysis of tumor antigen-specific T cells and antibodies in cancer patients treated with radiofrequency ablation. Int J Cancer.

[22] Gravante G, Sconocchia G, Ong SL (2009). Immunoregulatory effects of liver ablation therapies for the treatment of primary and metastatic liver malignancies. Liver Int.

[23] China Public Health Service’s Guide for Care and Use of Animals http://dwzx.suda.edu.cn/pages/vNone.aspx?id=543.

[24] Leytin VL, Misselwitz F, Lyubimova EV, Domogatsky SP (1989). The role of platelet prostanoids and dense granule compounds in initial attachment, spreading and aggregation of platelets on collagen substrates. Thromb Res.

[25] Cui J, Wang N, Zhao H (2014). Combination of radiofrequency ablation and sequential cellular immunotherapy improves progression-free survival for patients with hepatocellular carcinoma. Int J Cancer.

[26] Huang ZM, Li W, Li S (2013). Cytokine-induced killer cells in combination with transcatheter arterial chemoembolization and radiofrequency ablation for hepatocellular carcinoma patients. J Immunother.

[27] Pan CC, Huang ZL, Li W (2010). Serum alpha-fetoprotein measurement in predicting clinical outcome related to autologous cytokine-induced killer cells in patients with hepatocellular carcinoma undergone minimally invasive therapy. Chin J Cancer.

[28] Weng DS, Zhou J, Zhou QM (2008). Minimally invasive treatment combined with cytokine-induced killer cells therapy lower the short-term recurrence rates of hepatocellular carcinomas. J Immunother.

[29] Zerbini A, Pilli M, Penna A (2006). Radiofrequency thermal ablation of hepatocellular carcinoma liver nodules can activate and enhance tumor-specific T-cell responses. Cancer Res.

[30] Fagnoni FF, Zerbini A, Pelosi G, Missale G (2008). Combination of radiofrequency ablation and immunotherapy. Front Biosci.

[31] Zerbini A, Pilli M, Fagnoni F (2008). Increased immunostimulatory activity conferred to antigen-presenting cells by exposure to antigen extract from hepatocellular carcinoma after radiofrequency thermal ablation. J Immunother.

[32] Li X, Dai D, Song X (2014). A meta-analysis of cytokine-induced killer cells therapy in combination with minimally invasive treatment for hepatocellular carcinoma. Clin Res Hepatol Gastroenterol.

[33] Cui J, Wang N, Zhao H (2014). Combination of radiofrequency ablation and sequential cellular immunotherapy improves progression-free survival for patients with hepatocellular carcinoma. Int J Cancer.

[34] Weng DS, Zhou J, Zhou QM (2008). Minimally invasive treatment combined with cytokine-induced killer cells therapy lower the short-term recurrence rates of hepatocellular carcinomas. J Immunother.

[35] Motohashi S, Nakayama T (2009). Natural killer T cell-mediated immunotherapy for malignant diseases. Front Biosci (Schol Ed).

[36] Ayello J, van de Ven C, Cairo E (2009). Characterization of natural killer and natural killer-like T cells derived from ex vivo expanded and activated cord blood mononuclear cells: implications for adoptive cellular immunotherapy. Exp Hematol.

[37] Motohashi S, Nakayama T (2008). Clinical applications of natural killer T cell-based immunotherapy for cancer. Cancer Sci.

[38] Steinman RM, Hemmi H (2006). Dendritic cells: translating innate to adaptive immunity. Curr Top Microbiol Immunol.

[39] Thanendrarajan S, Nowak M, Abken H, Schmidt-Wolf IG (2011). Combining cytokine-induced killer cells with vaccination in cancer immunotherapy: more than one plus one?. Leuk Res.

[40] Märten A, Ziske C, Schöttker B (2001). Interactions between dendritic cells and cytokine-induced killer cells lead to an activation of both populations. J Immunother.

[41] Schmidt J, Eisold S, Büchler MW, Märten A (2004). Dendritic cells reduce number and function of CD4+CD25+ cells in cytokine-induced killer cells derived from patients with pancreatic carcinoma. Cancer Immunol Immunother.

[42] Li H, Ren XB, Zhang P, An XM, Liu H, Hao XS (2005). Dendritic cells reduce the number and function of CD4+CD25+ cells in cytokine-induced killer cells. Zhonghua Yi Xue Za Zhi.

